# Effects of the Japanese Herbal Medicine “Sho-saiko-to” (TJ-9) on Interleukin-12 Production in Patients with HCV-Positive Liver Cirrhosis

**DOI:** 10.1155/1999/62564

**Published:** 1999

**Authors:** Masayoshi Yamashiki, Akira Nishimura, Xian-Xi Huang, Tsutomu Nobori, Seigo Sakaguchi, Hiroyuki Suzuki

**Affiliations:** 1 Department of Laboratory Medicine Mie University School of Medicine Tsu Mie Japan; 2 Division of Gastroenterology University of Maryland Baltimore Maryland USA; 3 Division of Internal Medicine Suzuka General Hospital Suzuka Mie Japan; 4 Department of Gastroenterology Fukuoka University Chikushi Hospital Chikushino Fukuoka Japan; 5 Department of Laboratory Medicine Mie University School of Medicine 2-174 Edobashi Tsu Mie 514-0001 Japan

**Keywords:** interleukin- 12 (IL- 12), liver cirrhosis, Sho-saiko-to, herbal medicine, cytokine

## Abstract

Interleukin-12 (IL-12) is an important cytokine for maintainence of normal systemic defense and bioregulation. The Japanese herbal medicine Sho-saiko-to (TJ-9) has been administered to 1.5 million Japanese patients with chronic liver diseases. TJ-9 is known to significantly suppress cancer development in the liver and has macrobiotic effects. In the present study, we examined the *in vitro* production of IL-12 by circulating mononuclear cells from liver cirrhosis patients and the effects of TJ-9 on IL-12 production.

The monocyte/macrophage fraction and the lymphocyte fraction of peripheral blood were obtained from 11 HCV-positive liver cirrhosis patients and 12 healthy subjects. Interleukin-12 levels in the supernatants were measured using ELISA kits. The levels of IL-12 produced by the patients fractions were significantly lower than those produced by healthy subjects (*p* < 0.01, *p* < 0.05). However, when TJ-9 was added to the cultures, the IL-12 production levels in both cell fractions increased approximately three fold, and the levels from the monocyte/macrophage fraction were almost the same as those from healthy subjects. This effect of TJ-9 was attributable to two of its seven herb components, that is, *scutellaria root* and *glycyrrhiza root*. One possible mechanism for the macrobiotic effects of TJ-9 on liver cirrhosis patients may be the improvement in IL-12 production.


*Developmental Immunology*, 1999, Vol. 7(1), pp. 17-22
Reprints available directly from the publisher
Photocopying permitted by license only

© 1999 OPA (Overseas Publishers Association)
N.V. Published by license under the
Harwood Academic Publishers imprint,
part of Gordon and Breach Publishing Group
Printed in Malaysia

### Effects of the Japanese Herbal Medicine "Sho-saiko-to" (TJ-9) on Interleukin-12 Production in Patients with HCV-Positive Liver Cirrhosis

MASAYOSHI YAMASHIKI[ab*], AKIRA NISHIMURA[c], XIAN-XI HUANG[a], TSUTOMU NOBORI[a], SEIGO SAKAGUCHI[d] and HIROYUKI SUZUKI[b]

[a]*Department of Laboratory Medicine, Mie University School of Medicine, Tsu, Mie, Japan,* [b]*Division of Gastroenterology, University of Maryland, Baltimore, Maryland,* [c]*Division of Internal Medicine, Suzuka General Hospital, Suzuka, Mie, Japan and* [d]*Department of Gastroenterology, Fukuoka University Chikushi Hospital, Chikushino, Fukuoka, Japan*

*(Revised 21 May, 1998; In final form 05 August, 1998)*

Interleukin-12 (IL-12) is an important cytokine for maintainence of normal systemic defense and bioregulation. The Japanese herbal medicine Sho-saiko-to (TJ-9) has been administered to 1.5 million Japanese patients with chronic liver diseases. TJ-9 is known to significantly suppress cancer development in the liver and has macrobiotic effects. In the present study, we examined the *in vitro* production of IL-12 by circulating mononuclear cells from liver cirrhosis patients and the effects of TJ-9 on IL-12 production.

The monocyte/macrophage fraction and the lymphocyte fraction of peripheral blood were obtained from 11 HCV-positive liver cirrhosis patients and 12 healthy subjects. Interleukin-12 levels in the supernatants were measured using ELISA kits. The levels of IL-12 produced by the patients' fractions were significantly lower than those produced by healthy subjects ($p < 0.01$, $p < 0.05$). However, when TJ-9 was added to the cultures, the IL-12 production levels in both cell fractions increased approximately three fold, and the levels from the monocyte/macrophage fraction were almost the same as those from healthy subjects. This effect of TJ-9 was attributable to two of its seven herb components, that is, *scutellaria root* and *glycyrrhiza root.* One possible mechanism for the macrobiotic effects of TJ-9 on liver cirrhosis patients may be the improvement in IL-12 production.

*Keywords:* interleukin-12 (IL-12), liver cirrhosis, Sho-saiko-to, herbal medicine, cytokine

### INTRODUCTION

Interleukin-12 (IL-12) is known as the natural killer cell stimulating factor (Kobayashi et al., 1989), and its stimulatory effects are reported to be 100 to 1000 times more potent than that of IFN-α and IL-2 (Robertson et al., 1992; Chehimi et al., 1993b). The clinical application of IL-12 to cancer and immunodeficiency disease has been considered, because *in vitro* IL-12 improves the decreased cell-mediated immune responses and NK cell activity in patients with HIV infection (Chehimi et al., 1993a,

* Corresponding author. Present address: Masayoshi Yamashiki, Department of Laboratory Medicine, Mie University School of Medicine, 2–174 Edobashi, Tsu, Mie 514–0001, Japan. Tel.: +81–59–232–1111, Fax: +81–59–232–4049.

1994; Clerici et al., 1993), and when administered to mice transplanted tumors disappear (Brunda et al., 1994). Recently, we reported that IL-12 production capacity of circulating monocyte/macrophages correlated with prognosis of chronic hepatitis C patients (Yamashiki et al., 1997a).

Herbal medicines, which have been used in China for several thousand years, are increasingly being used among practioners of Western medicine in Japan. In particular, Sho-saiko-to (TJ-9; Xiao-Chai-Hu-Tang in Chinese), which has been used for pyretic diseases in China, has been officially approved in Japan as a medicine for hospital use with standard quality and quantity of each component, and it has now been prescribed to approximately 1.5 million patients with chronic viral liver diseases. There have been many Japanese studies on its clinical usefulness (Oka et al., 1984; Fujiwara et al., 1987; Tajiri et al., 1990) TJ-9 is well known to gradually improve the subjective symptoms and abnormal liver function of patients with chronic viral liver diseases. Recently, TJ-9 was reported to induce apoptosis *in vitro* of hepatocellular carcinoma cell lines (Yano et al., 1994), and a 5-year-observation of viral liver cirrhosis patients treated with or without TJ-9 demonstrated that TJ-9 significantly suppressed liver cancer development, and had macrobiotic effects (Oka et al., 1995). These findings elicited considerable responses from researchers. Some Japanese studies reported that TJ-9 enhances NK activities (Mizoguchi et al., 1986), and we have also demonstrated that TJ-9 induces various cytokines (Yamashiki et al., 1994, 1996, 1997b). Nowadays, TJ-9 has come to be considered as a biological response modifier.

Considering the importance of IL-12 in cancer and immunodeficiency diseases, the present study investigated the effects of TJ-9 on IL-12 production in the adherent cell (monocyte/macrophage) fraction and in the non adhered cell (lymphocyte) fraction of peripheral blood mononuclear cells (MNC) that were obtained from liver cirrhosis patients and healthy subjects. In addition, it compared the IL-12 production indices for each of the seven herb components of TJ-9.

### RESULTS

### Interleukin 12 production in the Monocyte/Macrophage Fraction

In the Mø fraction, IL-12 production levels were decreased in the patient group compared to healthy subjects: that is, in cultures with medium only, IL-12 production was 256 ± 54 pg/ml (mean ± SE) in patients, and 650 ± 139 pg/ml in a healthy subjects ($p < 0.05$, Table I). After SEB induction, IL-12 production levels were 1146 ± 181 pg/ml in patients and 2773 ± 491 pg/ml in a healthy subjects ($p < 0.01$). On the other hand, with TJ-9 in the cultures, the production levels in the both groups were increased to approximately three fold, that is, 765 ± 130 pg/ml in patients and 2183 ± 223 pg/ml in a healthy subjects.

### Interleukin 12 Production in the Lymphocyte Fraction

In the lymphocyte fraction, the IL-12 production levels in patients tended to be lower than in a healthy subjects, and after SEB induction, these levels were 310 ± 57 pg/ml in patients and 720 ± 132 pg/ml in healthy subjects ($p < 0.05$). With TJ-9, the production increased by three times or more in the patients, and by six times or more in the healthy subjects.

### Comparison of Seven Herb Components

In the comparison of the production index when a herb component, TJ-9, or SEB, was added to the Mø or lymphocyte fraction of healthy subjects, high scores were obtained for the *scutellaria root* (index score: 5.9), *glycyrrhiza root (3.7),* and *bupleurum root* (2.6) in the Mø fraction (Table II), and in the lymphocyte fraction, high scores were obtained for *scutellaria root* (9.4) and *glycyrrhiza root* (4.4). The index scores for the remaining five herb components were lower than 1.7.

TABLE I Interleukin 12 Production Levels in Patients with HCV-Positive Liver Cirrhosis

| *Reagents* | *Controls* | *Patients* | *p-value* |
|---|---|---|---|
| | In the Monocyte/Macrophage Fraction | | |
| Medium | 650 ± 139 | 256 + 54 | 0.0191 |
| SEB | 2773 ± 491 | 1146 + 181 | 0.0077 |
| LPS | 1413 ± 260 | 886 ± 286 | 0.1863 |
| TJ-9 | 2183 ± 225 | 765 ± 130 | 0.0004 |
| | In the Lymphocyte Fraction | | |
| Medium | 36.5 ± 5.7 | 31.3 ± 4.5 | 0.5082 |
| SEB | 720 ± 132 | 310 ± 57 | 0.0125 |
| Con A | 226 ± 31 | 181 ± 48 | 0.4372 |
| TJ-9 | 225 ± 59 | 97 ± 10 | 0.0546 |

Mean ± SE (pg/ml).
Controls: normal healthy subjects ($n$ = 12).
Patients: HCV-positive liver cirrosis patients ($n$ = 11).
$p$-value: obtained by unpaired $t$-test.
Medium: no stimulants.
SEB: staphylococcal enterotoxin B.
LPS: lipopolysaccharide.
TJ-9: Japanese herbal medicine "Sho-saiko-to".
Con A: concanavalin A.

TABLE II Interleukin 12 production index for each of Seven Herb Components

| *Reagent* | *Production Index* | |
|---|---|---|
| | In the Monocyte /ø Fraction | In the Lymphocyte Fraction |
| *Bupleurum root* | 2.64 ± 0.37 | 1.66 ± 0.31 |
| *Pinellia tuber* | 1.46 ± 0.15 | 1.48 ± 0.16 |
| *Scutellaria root* | 5.88 ± 1.01 | 9.42 ± 1.49 |
| *Jujube fruit* | 1.74 ± 0.33 | 1.36 ± 0.16 |
| *Ginseng root* | 1.40 ± 0.18 | 0.98 ± 0.05 |
| *Glycyrrhiza root* | 3.74 ± 0.77 | 4.42 + 1.46 |
| *Ginger rhizome* | 1.54 ± 0.29 | 1.02 ± 0.04 |
| TJ-9 | 5.13 ± 1.03 | 6.45 ± 1.41 |
| SEB | 4.75 ± 0.70 | 23.70 ± 5.60 |

Production index = (production level when a test substance was added to a culture) ÷ (production level when only medium control was added to a culture).
Mean ± SE.
ø: macrophage.
TJ-9: Japanese herbal medicine "Sho-saiko-to".
SEB: staphylococcal enterotoxin B.

### DISCUSSION

This *in vitro* study demonstrated that IL-12 production levels in both the Mø and lymphocyte fractions of the liver cirrhosis patients were significantly lower than those in the healthy subjects, and TJ-9 increased IL-12 production in both cell fractions of the patients to a level almost the same as those of healthy subjects. Interleukin 12 is produced by macrophages and B lymphocytes rather than T lymphocytes. The B/T ratio was higher in liver cirrhosis patients than in the healthy subjects (data not shown). Therefore, this suggests IL-12 production capacities of macrophage and B lymphocytes are impaired in liver cirrhosis patients. We consider that induction of IL-12 in chronic hepatitis C patients could be quite important to obtain a better therapeutic outcome (Yamashiki et al., 1997a).

The usual daily dose of TJ-9 is 7.5 g, and this contains 4.5 g of dried extract obtained from *bupleurum root, 7* g; pinellia tuber, 5 *g; scutellaria root,* 3 g; *jujube fruit,* 3 g; *ginseng root,* 3 g; *glycyrrhiza root,* 2 g; and *ginger rhizome,* 1 g. In the comparison of the production indexes, we used each herb component at the same concentration. We found that IL-12 was induced mainly by the *scutellaria root* and then by the *glycyrrhiza root.* These findings were similar to those found for the induction of TNF-α (Yamashiki et al, 1996). The major chemical content of *scutellaria root* is bicalin and that of *glycyrrhiza root* is glycyrrhizin. However, in an examination using these chemical compounds, no induction of IL-12 was observed (data not shown). Therefore, the IL-12 induction observed for the two herb components might be attributable to unknown chemical components.

TJ-9 strongly induces the production of various cytokines, in particular, IL-1β, IL-10, tumor necrosis factor alpha (TNF-α), and granulocyte colony-stimulating factor (G-CSF), by peripheral mononuclear cells (Yamashiki et al., 1994, 1996, 1997b). These effects are similar to those of LPS. To confirm that the observed *in vitro* TJ-9 effects were not induced by the mixed LPS into the TJ-9 bulk powder (which contains 0.00067% of LPS), we also examined IL-12 production by using reagents that were treated with taxol (an

LPS antagonist; Wako Pure Chemical Industries, Osaka). As a result, LPS-induced IL-12 production in the Mø fraction decreased by approximately 1/2 to 1/6 with taxol treatment, but IL-12 production induced by TJ-9 was not affected by taxol treatment. Therefore, we consider that increased IL-12 production in cultures with TJ-9 is not the result of mixed LPS in the culture but is specific to TJ-9. In addition, because the highest serum concentration of TJ-9 after 1 week of administration is presumed to be 200 to 300 μg/ml (Yano et al., 1994). the final concentration of TJ-9 used in this study (100 μg/ml) would be practical.

It is impossible to demonstrate the *in vivo* IL-12 induction by TJ-9 in patients with liver cirrhosis because the IL-12 level in the circulating blood is lower than minimum level measurable and also because the possibility of macrophages being activated and producing IL-12 without adhering to tissues is low. It would be worthwhile to investigate the changes in IL-12 producing cells of the organs such as the liver, spleen, lung, and lympho nodes from patients with liver cirrhosis, together with the expression of IL-12 messenger RNA in these organ tissues, and their changes after TJ-9 treatment.

Some of the advantages of herbal medicines are that their side effects are much milder than chemically produced drugs, and the herb moderately modifies the biological defense mechanism. However, at the same time, their moderate effects are a disadvantage: their efficacy in a short period of time is rather difficult to confirm, and many physicians, not only in Europe or the United States, but also in Japan, have doubted the efficacy of herbal medicines. In Japan, there have been 66 cases reported of interstitial pneumonia during TJ-9 treatment, mainly in hepatitis C patients. Although this incidence is quite low (far less than 0.01%) in the entire patients who received TJ-9 (approximately 1.5 million persons), 9 patients have died. This unfortunate event, however, also happens to indirectly suggest the effects of TJ-9 on the immunoregulation system.

Liver cancer is thought to occur within 10 years in 50% of viral liver cirrhosis patients. Induction of IL-12 may be useful to prevent cancer development, and IL-12 induction by TJ-9 could be an explanation for the macrobiotic effects of TJ-9 observed in the 5-year study of liver cirrhosis patients (Oka et al, 1995) In addition, because TJ-9 suppresses HIV proliferation *in vitro*, it may also be useful in the treatment of malignant diseases or immunodeficient diseases, including HIV infections (Buimovici-Klein et al., 1990). Through the clarification of the true immunological effects of herbal medicines, physicians may be able to obtain a novel and highly useful biological modifier, and herbal medicines could be an efficacious remedy for immune-related diseases as well as infectious diseases.

### MATERIALS AND METHODS

### Patients and Controls

Peripheral blood was collected from 11 liver cirrhosis patients and 12 healthy volunteers (university students or employees). They understood the purpose of this study, and consent was given for the blood sampling and its use in this study. The 11 patients were hepatitis C virus (HCV)-positive. The ages and sexes of the patients and the controls were roughly similar.

### Cell Preparation

As described in our previous studies (Yamashiki et al., 1994, 1996, 1997a, 1997b), heparinized peripheral venous blood was collected, and the mononuclear cell (MNC) fraction was obtained using the lymphocyte separation solution (Muto Pure Chemicals, Tokyo). The MNC fraction was washed 3 times with Roswell Park Memorial Institute (RPMI) medium 1640 (Gibco Laboratories, Grand Island, NY), and suspended at a density of $1 \times 10^6$cells/ml in the RPMI culture medium supplemented with 10% heat-inactivated fetal bovine serum (FBS, Gibco Laboratories) and two antibiotics (Flow laboratories, Irvine, Scotland), that is, penicillin 50 IU/ml (final concentration) and streptomycin 50 μg/ml. In all the following experiments except for washing, we used this MNC suspended RPMI medium (MNC suspension).

To obtain cell fractions, the wells of 24-well culture plates (Becton Dickinson Labware, Lincoln Park, NJ) were coated with heat-inactivated human serum type AB, then 1 ml of the MNC suspension was poured into the wells, and incubated in 5% $CO_2$ -in-air for 60 min at 37°C. The non adhered cells were obtained as the lymphocyte fraction. The adhered cells were washed twice with RPMI-1640. These cells were then detached with 0.02% ethylenediamine tetraacetic acid (EDTA) solution in 0.85% saline (Flow Laboratories), and used as the Mø fraction. Cell surface markers were examined using a flow cytometer (FACScan, Beckton Dickinson, San Jose, CA), and CD14-positive cells were found to be 1% or less in the lymphocyte fraction, and 90% or more in the Mø fraction.

### Reagents

The bulk powder of TJ-9 and its seven herb components were supplied by Tsumura & Co. (Tokyo). Each drug was dissolved into distilled water by gently shaking the tube for 2 h at 37°C, and then centrifuging twice to remove precipitates. The solution was passed through a filter unit (Millipore Products Division, Bedford, MA) for sterilization, and then diluted to the final concentration (100 μg/ml) with the RPMI-1640 solution supplemented with the antibiotics. Stimulants used in this study were staphylococcal enterotoxin B (SEB, Wako Pure Chemical Industries, Osaka), concanavalin A (conA, Sigma Chemical, St. Louis) and lipopolysaccharide (LPS, Sigma).

### Cell Culture

The Mø suspension was adjusted to $1.5 \times 10^5$ cells /ml and the lymphocyte suspension to $8.5 \times 10^5$ cells/ml with the previously mentioned culture medium. To each well of a 24-well culture plate, 980 μl of either suspension and 20 μ l of a single test substance or RPMI-1640 medium was added. The plate was cultured with 5% $CO_2$-in-air at 37°C for 48 h, and then the supernatants were collected by centrifugation. The final concentration of the drugs and reagents were as follows: 100 μg/ml for TJ-9 and the seven herb components; 5 μg/ml for SEB and conA; and 5 ng/ml for LPS.

### Cytokine Level

Interleukin 12 levels in the supernatant were measured using ELISA kits (T Cell Diagnostics, Woburn, MA). The IL-12 production with each of the seven herb components was examined using the Mø and lymphocyte fractions obtained from five healthy subjects. The results were expressed in terms of the production index, which was defined as the level of IL-12 produced in the presence of a drug or reagent divided by the level produced with culture medium alone.

### Statistical Analysis

Statistical analyses on the differences of production levels between the healthy subjects and liver cirrhosis patients were carried out using non paired t-test (Student's t-test and Welch's *t*-test).

***Acknowledgements***
We would like to express our sincere appreciation to Professor Stephen P. James, Division of Gastroenterology, University of Maryland, for his valuable advice and comments, and Mr. Kiyotaka Matsushita, Mr. Hiroyuki Ohtaki, and Mr. Akiharu Nakai, Tsumura & Co., for their extensive assistance.

***References***

Brunda M.J., Luistro L., Warrier R.R., Wright R.B., Hubbard B.R., Mirphy M., Wolf S.F., and Gately M.K. (1994).Antitumor and antimetastatic activity of interleukin 12 against murine tumors. J. Exp. Med. **178**: 1223–1230.

Buimovici-Klein E., Mohan V., Lange M., Fenamore E., Inada Y., and Cooper L.Z. (1990). Inhibition of HIV replication in lymphocyte culture of virus-positive subjects in the presence of Sho-saiko-to, an oriental plant extract. Antiviral Res. **14**: 279–286.

Chehimi J., Starr S.E., Frank I., D'Andrea A., Ma X., MacGregor R.R., Sennelier J., Trichieri G. (1994). Impaired interleukin 12 production in human immunodeficiency virus-infected patients. J. Exp. Med. **179**: 1361–1366.

Chehimi J., Starr S., Frank I., Rengaraju M., Jackson S.J., Llanes C., Kobayashi M., Perussia B., Young D., Nickbarg E., Wolf S.F., Trichieri G. (1993a). Natural killer cell stimulatory factor

(NKSF) increases the cytotoxic activity of NK cell from both healthy donors and HIV-infected patients. J. Exp. Med. **175**: 789–796.

Chehimi J., Valiante N.M., D'Andrea A., Rengaraju M., Rosade Z., Kobayashi M., Perussia B., Wolf S.F., Starr S.E., Trichieri G. (1993b) Enhancing effect of natural killer cell stimulatory factor (NKSF/interleukin-12) on cell-mediated cytotoxicity against tumor-derived and virus-infected cells. Eur. J. Immunol. **23**: 1826–1830.

Clerici M., Lucey D.R., Berzofsky J.A., Pinto L.A. Wynn T.A., Blatt S.P., Dolan M.J., Hendrix C.W., Wolf S.F., Shearer G.M. (1993). Restoration of HIV-specific cell-mediated immune responses by interleukin 12 in vitro. Science **262**: 1721–1724.

Fujiwara K., Ohta Y., Ogata I., Hayashi S., Sato Y., Oka Y., and Yamada S. (1987). Treatment trial of traditional Oriental medicine in chronic viral hepatitis. In New trends in Peptic Ulcer and Chronic Viral Hepatitis: Part II. Chronic hepatitis. Ohta Y., ed (Tokyo: Excerpta Medica), pp. 141–146.

Kobayashi M., Fitz L., Ryan M., Hewick R.M., Clark S.C., Chan S., LoudonR., Sherman F., Perussia B., and Trichieri G. (1989). Identification and perification of natural killer cell stimulatory factor (NKSF), a cytokine with multiple biologic effects on human lymphocytes. J. Exp. Med. **170**: 827–845.

Mizoguchi Y., Fujinobu Y., Kobayashi Y., Yamamoto S., and Morisawa S. (1986). Effects of Sho-saiko-to on natural killer (NK) cell activity. Wakan Pharmaceutical Society J. **3**: 184–189

Oka H., Fujiwara K Oda T. (1984). Xiano-Chai-Hu-Tang and Gui-Zhi-Ling-Wan for treatment of chronic hepatitis. In Recent Advances in Traditional Medicine in East Asia, Oda T., Needham J., Otsuka Y., Guo-bin L., Eds. (Amsterdam: Excerpta Medica), pp. 232–237.

Oka H., Yamamoto S., Kuroki T., Harihara S., Marumo T., Kim S.R., Monna T., and Kobayashi K. (1995). Prospective study of chemotherapy of hepatocellular carcinoma with Sho-saiko-to (TJ-9). Cancer **76**: 743–749.

Robertson M.J., Soiffer R.J., Wolf S.F., Manley T.J., Donahue C., Young D., Herrmann S.H., and Rits J. (1992). Responses of human natural killer (NK) cell to NK cell stimulatory factor (NKSF): Cytolytic activity and proliferation of NK cells are differentially regulated by NKSF. J. Exp. Med. **175**: 779–788.

Tajiri H., Kozaiwa K., Osaki Y., Hamamoto T., Miki K., Shimizu K., and Ida S. (1990). The study of the effect of Sho-saiko-to on HBe Ag clearance in children with chronic HBV infection and with abnormal liver function test results. Acta Paediatr. Jpn **94**: 1811–1815

Yamashiki M., Nishimura A., Nomoto M., Nakano T., Sato T., Suzuki H., Zheng Q.-X., Klapproth J.-M., James S.P., Kosaka Y. (1994). Effects of Japanese herbal medicine "Sho-saiko-to" on *in vitro* production of peripheral blood mononuclear cells. Drug Develop. Res. **31**: 170–174.

Yamashiki M., Nishimura A., Sakaguchi S., Suzuki H., and Kosaka Y. (1996). Effects of the Japanese herbal medicine "Sho-saiko-to" as a cytokine inducer. Environ. Toxicol. Pharmacol. **2**: 301–306

Yamashiki M., Nishimura A and Suzuki H. (1997a). Prognosis of chronic hepatitis C patients correlates with circulating monocyte/macrophage function. Clin. Sci. **93**: 381.

Yamashiki M., Nishimura A., Sakaguchi S., Suzuki H., Kosaka Y. (1997b). Effects of the Japanese herbal medicine "Sho-saiko-to" (TJ-9) on in vitro interleukin-10 production by peripheral blood mononuclear cells of patients with chronic hepatitis C. Hepatology **25**: 1390–1397.

Yano H., Mizoguchi A., Fukuda K., Haramaki M., Ogasawara S., Momosaki S., Kojiro M (1994). The herbal medicine Sho-saiko-to inhibits proliferation of cancer cell line by inducing apoptosis and arrest at the G0/G1 phase. Cancer Res. **54**: 448–454.

